# Extreme heat and pediatric health in a warming world: a space-time stratified case-crossover investigation in Ontario, Canada

**DOI:** 10.1186/s12940-025-01153-y

**Published:** 2025-06-07

**Authors:** Hallah Kassem, Eric Lavigne, Kate Weinberger, Michael Brauer

**Affiliations:** 1 School of Population and Public Health, University of British Columbia, 2206 East Mall, Vancouver, BC V6T 1Z3 Canada; 2 School of Epidemiology and Public Health, 600 Peter Morand Crescent, Ottawa, ON K1G 5Z3 Canada; 3 Environmental Health Science and Research Bureau, Health Canada, Ottawa, ON K1A0K9 Canada; 4 ICF, 1200 Sixth Avenue Suite 1800, Seattle, WA 98101 USA; 5 School of Public Health, University of Washington, Seattle, United States

**Keywords:** Environmental epidemiology, Pediatric health, Climate change, Extreme heat, Hospital admissions, Emergency department

## Abstract

**Background:**

Globally, climate change is causing frequent and severe extreme heat events (EHEs). A large body of literature links EHEs to multiple health endpoints. While children’s physiology and activity patterns differ from those of adults in ways that are hypothesized to increase susceptibility to such endpoints, research gaps remain regarding the specific impacts of EHEs on child health. This study evaluated pediatric emergency healthcare utilizations associated with EHEs in Ontario.

**Methods:**

Applying a space-time stratified case-crossover design, associations between EHEs (same-day or lagged exposure to 2 consecutive days of daily maximum temperatures above percentile thresholds) and 15 causes of pediatric emergency healthcare use in Ontario, Canada from 2005 to 2015 were analysed using conditional quasi-Poisson regression. In primary analyses, EHEs were defined as two or more consecutive days with temperatures above the 99th percentile of temperature within each respective forward sortation area (FSA). Emergency healthcare use was measured using hospital admissions as an indicator of severe outcomes, and emergency department (ED) visits as a sensitive measure of outcomes.

**Results:**

Relative to non-EHE days, EHEs increased the rates of pediatric hospital admissions for respiratory illnesses by 26% (95% CI: 14-40%), asthma by 29% (16-44%); infectious and parasitic diseases by 36% (24-50%), lower respiratory infections by 50% (36-67%), and enteritis by 19% (7-32%). EHEs also increased the rates of ED visits for lower respiratory infections by 10% (0-21%), asthma by 18% (7-29%), heat-related illnesses by 211% (193-230%), heatstroke by 590% (550-622%), and dehydration by 35% (25-46%), but not for other causes. Admissions and ED visits due to injuries and transportation related injuries were negatively associated with EHEs. Neither all-cause hospital admissions nor ED visits were associated with EHEs.

**Conclusions:**

In Ontario, EHEs decreased the rates of pediatric emergency healthcare utilization for injuries and increased the rates of respiratory illnesses, asthma, heat-related illnesses, heatstroke, dehydration, infectious and parasitic diseases, lower respiratory infections, and enteritis. Tailored policies and programs that reflect the specific heat-related vulnerabilities of children to respiratory and infectious illnesses are warranted in the face of a rapidly warming climate.

**Supplementary Information:**

The online version contains supplementary material available at 10.1186/s12940-025-01153-y.

## Background

Climate change threatens the health of populations through a myriad of hazards including wildfires, droughts, famines, drinking water contamination, vector-borne diseases, and extreme heat events (EHEs). According to Canada’s Changing Climate Report, Canada is warming at twice the average rate globally [1]. The annual number of extremely hot days is estimated to double in Canada over the next 30 years [2]. Definitions of an EHE vary by region but are generally defined as 2 or more consecutive days with average temperatures significantly greater than typical for the region [3]. In Canada, this temperature is typically around 30 °C (between 28 °C and 35 °C), although it varies between and within provinces and territories [4]. Heat can directly impact multiple organ systems, worsen existing conditions, and increase the risk of injuries by impacting behaviour [5–8]. EHEs can also overwhelm healthcare services and infrastructure such as the supply of electrical power and water, as the rate of resource use exceeds system capacities; this can increase risk of transmission of water- and food-borne illnesses [5, 7, 9, 10]. 

While the epidemiologic literature on heat has focused primarily on adults, children may exhibit unique effects. Within the literature on EHEs and pediatric health outcomes, studies have typically used coarse age groups for children under 18. Children’s activity patterns and dependence on caregivers may also heighten their susceptibility as they spend more time outside and are unable to respond to heat themselves [11]. Compared to adults, children have a higher surface area to mass ratio, higher temperature at which sweating begins, lower sweating capacity, lower blood volume and lower cardiac output [12, 13]. These physiological factors combine to increase strain on circulatory systems and decrease ability to thermoregulate [12]. Only two studies have been conducted in Canada which examined solely hospitalizations due to drownings [14] and emergency department (ED) visits due to respiratory and infectious diseases [15].

In part due to lack of research specific to the impact of EHEs on children, current heat-related public health interventions may not be optimally tailored to the unique needs of children. For example, the temperature thresholds used for Ontario’s heat warning criteria are based on increases in all-cause mortality of the general population [4]. The aim of this study was to identify specific pediatric uses of emergency healthcare associated with EHEs among children in Ontario.

## Methods

### Population

The population in this study was children ages 0–18 years in Ontario who were admitted to a hospital for urgent (i.e. non-elective) care or to an ED during warm months (May-September) between 2005 and 2015, thus 10 years of data were used. Age was categorized into “0–4”, “5–12” and “13–18” years. These categories were selected to facilitate comparisons with similar studies [15–19]. Sex was categorized as male or female; records with missing responses for sex were included in analyses that were not stratified by sex. Residential locations of study participants were examined at the resolution of the Forward Sortation Area (FSA) boundaries. FSAs are the first three digits of a postal code and reflect the part of the province, whether the area is rural or urban and the specific region, though do not reflect the population or geographic size [20]. 

### Outcome measures

This study examined two measures of emergency healthcare utilization which may reflect conditions of varying severity: hospital admissions and ED visits. A child with a severe condition requiring longer-term treatment, for example a severe asthma attack, would be admitted to the hospital following an ED visit. Conversely, conditions needing only short-term emergency healthcare assessment, for example receiving a prescription for an antibiotic to treat otitis, would need only to be seen in the emergency department without escalation to hospital admission. The inclusion of both datasets in this study allows for the comparison of type of healthcare utilization by condition.

Several causes (all-cause, respiratory, asthma, injury, heat-related, heatstroke, dehydration, renal disease, infectious diseases, otitis, enteritis) were defined a priori based on (a) literature [11, 17, 19, 21–25], and (b) causes of pediatric emergency healthcare use not previously addressed in a Canadian context (all-cause, asthma, injury, falls, transportation-related injuries, heat, heatstroke, dehydration, renal disease, otitis, enteritis, lower respiratory infections) that we hypothesized as potentially being associated directly or indirectly with EHEs. Deidentified hospital admission data from the Discharge Abstract Database (DAD) and ED visit data from the National Ambulatory Care Reporting System (NACRS), national databases of healthcare utilization by province, were classified using the medical classification list of the World Health Organization’s (WHO) 10th revision of the International Statistical Classification of Disease and Related Health Problems (ICD-10), shown in Table 1. ICD-10 codes reached complete implementation in Ontario in 2002 [26]. The ICD-10 codes of the primary diagnoses were used in defining outcome. However, ICD-10 codes for external causes like drownings and falls are specified in secondary diagnosis fields; so, these external causes were defined using ICD-10 codes in either primary or secondary fields. These data provided counts of ED visits and hospital admissions by cause, date, age, sex, and FSA. No personal identifying information on each presentation were provided therefore it was not possible to link the ED visit and hospital admission data to each other, rather analyses of each were conducted separately.

**Table 1:** Outcome measures and corresponding ICD-10 codes

Outcome	ICD-10 Code(s)
Respiratory	J00-J99
Asthma	J45
Injury	S00-T66, T68-88
Drowning	V90, V92, W67-W70, W73, W74
Falls	W00-W19
Transportation	V01-V99
Heat	T67, E86, E87
Heatstroke	T67
Dehydration	E86-87
Renal	N00-N399
Inf/Parasitic	A00-B99
Otitis	H60, H65-67
Enteritis	A00-A09
Lower Resp	J12-J18, J20-J22

### Exposure measures

EHEs are generally defined as consecutive days with daily temperatures significantly greater than expected for the given location [3]. Commonly, temperature thresholds are set at the regional 95th [25, 27–29], 97.5th [27], and/or the 99th [29–33] percentile of the location-specific seasonal temperature distribution. Similarly, this study used percentile-based thresholds of daily maximum temperature for each FSA during warm months (May to September) averaged over the 10-year study period (2005–2015). The primary analysis defined EHEs as two consecutive days above the 99th percentile, the most restrictive definition of a heatwave. Broader definitions were used in sensitivity analyses using the 97.5th and 95th percentiles of temperature, and 1- or 2-day lag periods since lagged effects have been found in similar studies [10, 33–37]. 

Sensitivity analyses using the 97.5th and 95th percentiles evaluated variation in temperatures at which outcomes are associated with extreme heat. These analyses may also address variations in the objective temperature of the relative 99th, 97.5th and 95th temperature percentiles averaged over the 10-year study period. 1- and 2-day lag analyses were included to assess outcomes that result 1 or 2 days after 2 consecutive days of extreme heat exposure.

Heat warnings in Ontario are issued by Environment and Climate Change Canada (ECCC) at temperatures akin to the regional 95th percentile of temperature found in this study [4]. Relative humidity inhibits the thermoregulatory effect of sweating by reducing the evaporative capacity of the environment. For this reason, relative humidity, rather than absolute humidity, was evaluated within a sensitivity analysis, as evaluated in similar analyses [14, 15, 24, 38, 39]. Exposure measures of all analyses are summarized below in Table 2.

**Table 2:** Exposure measures and corresponding definitions

Exposure	Definition
Primary Analysis	
99th percentile Lag 0	Same day exposure to 2 consecutive days with daily maximum temperature above the 99th percentile of temperature within an FSA
Sensitivity Analyses	
Relative humidity	Same day exposure to 2 consecutive days with daily maximum temperature above the 99th percentile of temperature within an FSA, controlling for relative humidity
Lag 1	1 day lagged exposure to 2 consecutive days with daily maximum temperature above the 99th percentile of temperature within an FSA
Lag 2	2 day lagged exposure to 2 consecutive days with daily maximum temperature above the 99th percentile of temperature within an FSA
97.5th percentile	Same day exposure to 2 consecutive days with daily maximum temperature above the 97.5th percentile of temperature within an FSA
95th percentile	Same day exposure to 2 consecutive days with daily maximum temperature above the 95th percentile of temperature within an FSA

### Analytic approach

#### Study design

This study utilized a space-time-stratified case-crossover study design. Stratification of space was done at the residential FSA level, and stratification of time was done by day-of-week. The case-crossover design, as an alternative to time series regression, is commonly used in environmental epidemiology studies of associations between short-term exposures and outcomes [40] by comparing outcomes in individuals when exposed and unexposed [41]. This study applied this design by comparing daily counts of pediatric emergency healthcare utilization on EHE days to 3 or 4 control days (one per week) on the same day of week in the same month and year within the same FSA, effectively comparing healthcare utilization within the same population. A visual illustration of the study design can be found in the supplementary material: Additional_File_2.

By matching counts on exposed days to unexposed days drawn from the same populations, confounding by variables that do not vary week to week (ex. age, sex, SES) is eliminated by design [42]. Risk of confounding is thereby limited to variables that change over short periods of time (e.g. humidity). Similarly, since population sizes of FSAs do not significantly change week to week there is no need to incorporate population offsets in the models. In case-crossover studies, control days are often selected unidirectionally (control days are either before or after case day) or bidirectionally (control days are equally split before and after case day). These selection methods, however, could introduce risk of biases from time-trends in exposure or outcome [43]. The time-stratified case-crossover design of this study removes patterns in control days (control days may be before and/or after case day), thus avoiding risk of time-trend biases [44, 45]. 

#### Analysis

Daily 1-squared-kilometer gridded estimates of temperature were computed by Daymet, supported by NASA, and were aggregated to the FSA level [46]. A custom QGIS [47] plugin integrating the Google Earth Engine (GEE) python API was used to automate the extraction of the Daymet grid cell-level data [48]. Daily temperature and water pressure values were averaged across inhabited land within each FSA using the GEE mean reducer algorithm. Given the extensive heat sink coverage of water and forest in Ontario, it was important to identify inhabited land. Inhabited land, defined as having a population density of 0.4 or more people per square kilometre, was identified using ecumene boundaries retrieved from Statistics Canada [49]. After restricting to these ecumene, the resulting polygons were used to define each FSA. The boundary of each FSA’s polygon may include 100% of some gridded cells and only part of other cells. Thus, to calculate the average temperature within an FSA, the Daymet daily temperature in each gridded cell was weighted corresponding to that cell’s pixel fraction inside the FSA polygon using the ee.Reducer.mean() parameter [48]. The resulting dataset contained the FSA (of child’s residence); maximum, minimum and average daily temperatures in °C; water vapour pressure in kPa; and relative humidity (using Bolton’s Equation).

Temperature equivalents of the 99th, 97.5th or 95th percentiles were identified in each FSA. A binary (0/1) indicator variable was created to identify days which met the EHE definition for each FSA (see Table 2). Days on which the maximum temperature exceeded the threshold of the respective FSA *and* was preceded by a day on which the maximum temperature also exceeded the threshold was designated with a 1 to indicate it as an EHE day. All other days were considered non-EHE days (designated with a 0). Using a similar binary indicator variable, lag 1- and 2-days were identified as 1 or 2 days after an EHE day.

Using date and FSA, the data merge was conducted with 100% linkage. The percentile-specific data frames were fit in generalized nonlinear models (gnm) of conditional quasi-Poisson regression in R using the “gnm” package [50]. Relative rate ratios (RR) and 95% confidence intervals (CI) were calculated for each outcome comparing 1-day rates of pediatric emergency healthcare utilization outcomes on EHE days relative to those on non-EHE days [40]. Associations were also estimated by sex and 5-year age groups.

## Results

**Fig. 1:**
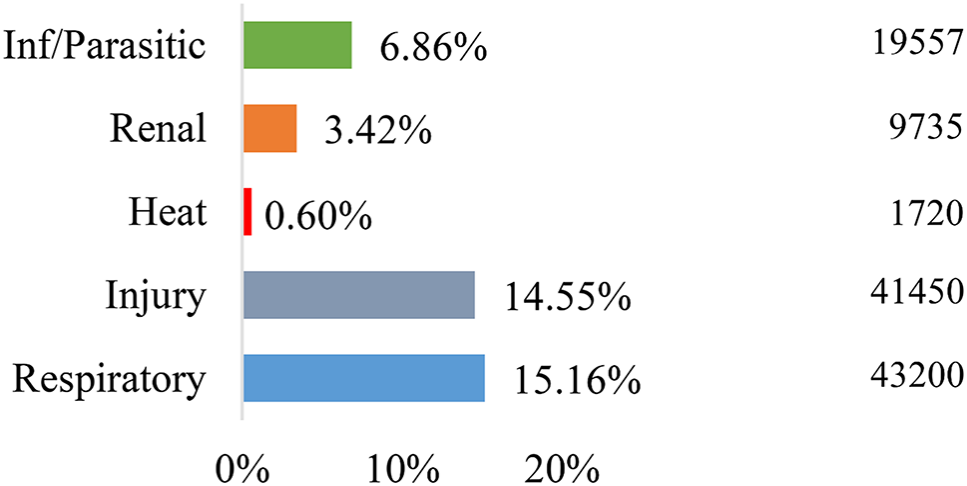
Proportion of hospital admissions by cause (*N* = 284,939)

**Fig. 2:**
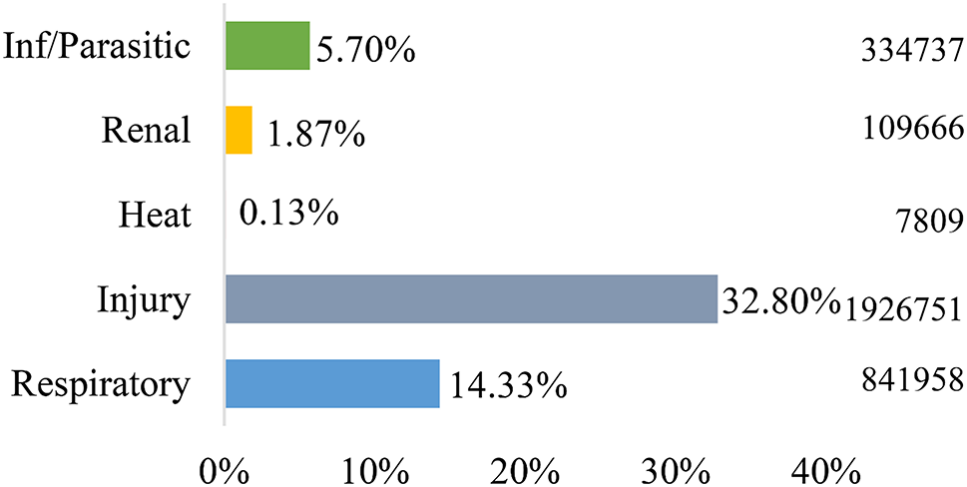
Proportion of ED visits by cause (*N* = 5,875,119)

284,939 all-cause hospital admissions and 5,875,119 all-cause emergency department visits were included in this study. Of the 5 general causes included in this analysis, injuries and respiratory illnesses were the most common causes of both hospital admissions (14.55% and 15.16%, respectively) and ED visits (32.80% 14.33%, respectively), as shown in Figs. 1 and 2.

To maintain precision of reported results, ED visits due to drowning, and hospital admissions due to drowning, heat, heatstroke, dehydration, and otitis were omitted as these outcomes occurred fewer than 20 times on EHE days during the study period.

In contrast to ED visits, individuals who sought emergency care may be admitted to hospitals if they present with symptoms requiring specialized care or extended observation. The fewer counts of hospital admissions compared to ED visits likely reflects the relative rarity of ailments requiring escalation from an ED visit to an admission. Consequently, the results herein should be interpreted considering *both* outcome measures and in consideration that differences observed between hospital admissions and ED visits may reflect the nature of the care required.

**Fig. 3:**
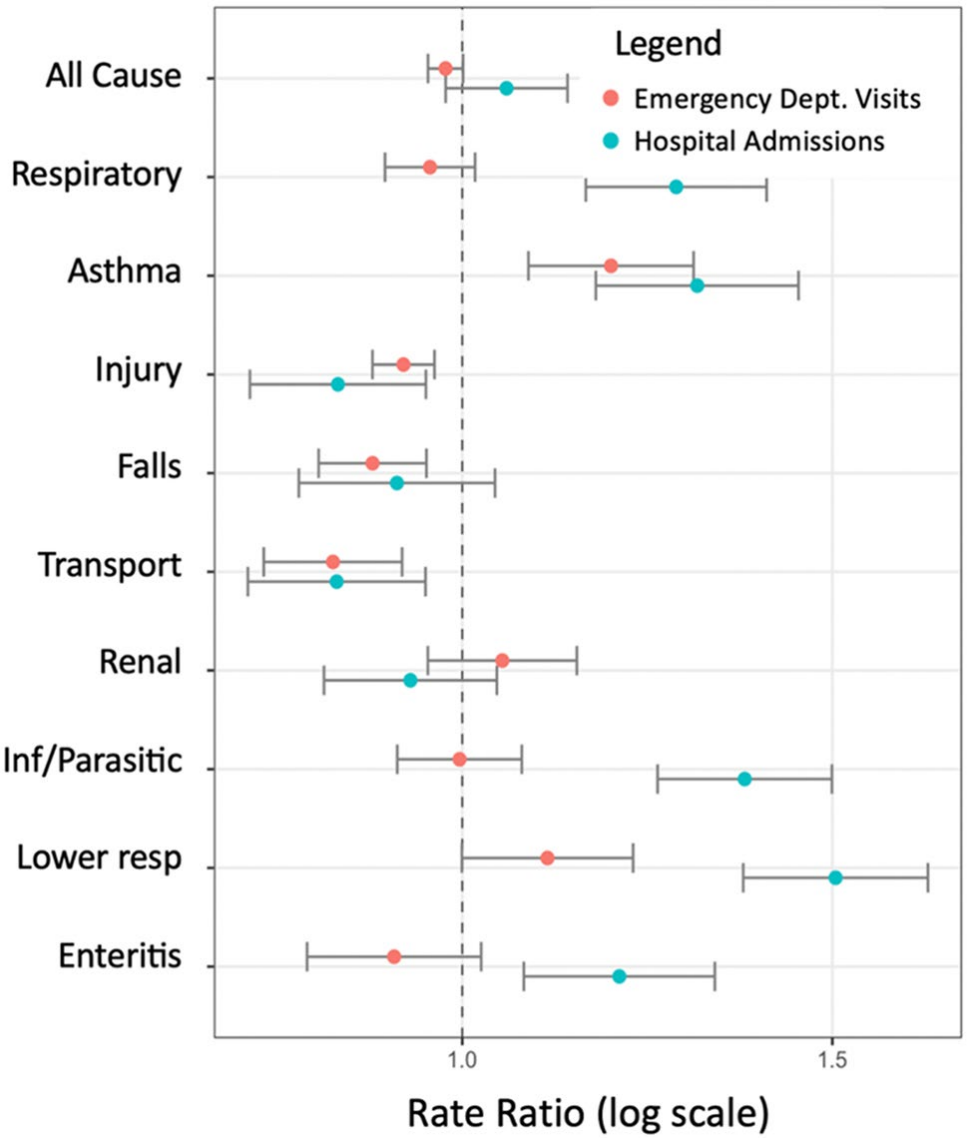
Rate ratios of pediatric emergency healthcare utilization associated with extreme heat events

Relative to non-EHE days, during EHEs statistically significant increases in daily rates were observed for hospital admissions due to respiratory illnesses, asthma; infectious and parasitic diseases, lower respiratory infections, and enteritis (Fig. 3). Similarly, on EHE days, rate increased for ED visits due to asthma; heat-related illnesses, heatstroke, dehydration, and lower respiratory infections. In contrast, decreases were observed on EHE days for both hospital admissions and ED visits for injuries and transportation-related injuries, as well as ED visits due to falls.

Associations observed in the primary analyses were consistent when adjusting for relative humidity (see tables A4 and A5). Associations were generally attenuated in the days following an EHE (lag 1 or 2) for most outcomes, however, hospital admissions and ED visits due to enteritis were highest the day after an EHE (lag 1). Similarly, 2 days after an EHE (lag 2) RR of renal and infectious and parasitic disease hospital admissions was highest. Associations were generally strongest in the primary analysis and approached the null in sensitivity analyses with lower threshold temperatures. For example, asthma admissions were associated with a 29% (95%CI: 16%, 44%) increased RR when EHEs were defined at the 99th percentile of temperature, 8% (2%, 15%) at the 97.5th, and a null effect (-7%, 0%) at the 95th; conversely, EHEs were associated with a *decreased* RR of injury admissions by 13% (21%, 4%) at the 99th percentile, 7% (11%, 2%) at the 97.5th and a null effect (4%, -2%) at the 95th. These patterns suggest rates of pediatric emergency healthcare use increase with increasing temperature.

As shown in Tables A6 and A7, EHEs had the fewest positive associations with hospital admissions or ED visits in children in the 5-12-year age group. It follows, then, that most all-cause admissions and ED visits were found in the 0–4 age category (46.96% and 34.76%, respectively) and the 13–18 age category (29.46% and 33.42%, respectively), with the fewest in the 5–12 age category (23.58% and 31.82%, respectively).

In sex-stratified analyses, presented in Tables A8 and A9, more harmful associations were found for hospital admissions than ED visits, and in females than males. When compared to hospitalizations, ED visits had fewer associations of elevated RR overall and far fewer that were shared by both sexes. During EHEs, both sexes exhibited higher RR of hospitalizations due to respiratory illness, asthma; infectious and parasitic diseases, lower respiratory infections and enteritis, and ED visits due to asthma, heat and heatstroke. The sexes differ, however, in associated rates of injury, falls, transportation-related injury and renal disease for which males’ RR decreased while females’ RR increased or saw no change during EHEs. During EHEs, hospital admissions for injuries and transportation-related injuries increased in females and decreased in males.

## Discussion

Many results of this study align with hypothesized effects and relationships based on biological plausibility, and evidence from similar research on respiratory illness [38, 51], asthma [37], enteritis [18], and direct heat-related illnesses [17, 18, 25, 28, 34, 51]. In the primary analysis, EHEs were found to increase the rate of hospital admissions due to general respiratory illnesses and asthma; general infectious and parasitic diseases, lower respiratory infections, and enteritis. Similarly, rates of ED visits due to asthma; heat-related illnesses, heatstroke, dehydration, and lower respiratory infections were positively associated with EHEs.

Interestingly, EHEs were found to reduce rates of hospital admissions and ED visits for general injuries and transportation related injuries, and ED visits due to falls. This novel finding contrasts with those of similar studies in settings including New York City [52, 53] and England [54] in which injuries amongst children were found to increase during EHEs. This may reflect the efficacy of heat warnings in mitigating effects of EHEs on child health through activity modifications like abstaining from sports and physical activities, for example. The same theory may help explain why sex stratification showed that during EHEs males reduced rates of injuries, falls, and transportation-related injuries as well as hospitalizations due to renal disease. This protective effect contrasts with results of a study in New York City which found the highest risk of unintentional injury among males ages 5 to 9 years old [52]. When stratified by age, the 0–4 and 13–18 year age groups exhibited the most positive associations. Previous studies have also found that, when compared to older age groups, children under 5 have higher rates of emergency department visits [53], asthma [37], respiratory illness [38, 51], and renal diseases [51]. This may be due to heightened caution for the 0–4 age group by caregivers and healthcare providers increasing the likelihood of an ED visit patient being admitted, and due to the 13–18 age group being more physically active and independent which can heighten susceptibility to EHEs.

Adjusting for relative humidity did not alter primary results. Associations, either positive or negative, were often strongest in the primary analysis and approached the null in sensitivity analyses with lower threshold temperatures and lag days from exposure. This monotonically decreasing trend effect demonstrates an exposure-response relationship between heat and rates of pediatric emergency healthcare. However, although RR of most outcomes were generally highest on same-day exposure to EHEs, hospital admissions and ED visits due to enteritis were highest the day after an EHE (lag 1), reflecting typical incubation periods of common foodborne and waterborne pathogens [55] including nontyphoidal *Salmonella* spp. (12–96 h) [56], norovirus (12–48 h) [57], and *Campylobacter* spp. (2–4 days) [58]. Similarly, RR of renal and infectious and parasitic disease hospital admissions were highest 2 days after an EHE (lag 2), possibly reflecting the cumulative effects of days of dehydration on renal and immune system function.

EHEs do not have a universal definition. Rather, they vary in threshold, temperature, and length making relative each of the terms “extreme”, “heat” and “event”. This consequently complicates comparisons. When aptly compared to the results of primary or sensitivity analyses herein, similar literature has made similar findings. Pediatric hospital admissions due to respiratory illnesses [25, 38], asthma [37], and heat [25] were positively associated with EHEs in Australia, the US, and China. It is also important to note that studies used varied age categories for children [15, 18], including several which used coarse age categories of 0–14 and 15–64 years [51], or 0–4 and 5–65 [17].

In two Ontario hospitals, Wilk et al. found pediatric infectious and respiratory diseases ED visits were positively associated with EHEs. This is the only prior study of pediatric ED visits in Canada, and it included only these two outcome measures. Respiratory illnesses [51], asthma [34], heat-related illnesses [26, 29, 44, 45], dehydration [17, 34, 51, 59], renal diseases [25], otitis [18], and bacterial enteritis [18, 34] have been positively associated with EHEs in studies outside of Canada. Of these outcomes, our study similarly found that EHEs increased rates of ED visits for asthma, heat-related illnesses, and dehydration in primary analysis, as well as with renal diseases and otitis in 1-day lag, and renal diseases under the 97.5th and 95th percentile definitions.

To date, this study was the largest and most comprehensive examination globally of EHEs and pediatric healthcare use, and the first study in Canada to assess associations of EHEs and pediatric hospital admissions. The data spanned the warm months of 10 years, including 284,939 hospital admissions, and 5,875,119 ED visits. The large scale of this analysis increases the power and representativeness of the study, especially for general, broadly defined causes of admissions and ED visits such as respiratory disease and injury. Given that Ontario provides universal free healthcare to residents and reports all admissions and ED visits to NACRS and DAD, the data used in this study is comprehensive and includes all pediatric cases. The space-time stratified case crossover design applied in this analysis controls for characteristics that do not vary over time or vary only slowly (e.g., age, race, sex) and for time-trend and seasonal patterns. Conditional quasi-Poisson regression, common in similar studies [15, 60–66], was applied instead of the traditionally used conditional logistic regression because it is simpler and faster to code, and allows for adjusting for overdispersion and auto-correlation [40]. Poisson distribution models rate counts given exposure, whereas conditional logistic regression models odds of a binary outcome given a one unit increase in exposure. To create a binary outcome to use conditional logistic regression, the data would need to be expanded such that each stratum contains only one case, or semi-expanded such that each stratum contains only one case *day* and was weighted by the number of cases that day. This study also benefits from the application of Daymet daily estimates of temperature aggregated to residential FSA, which are a more accurate measurement of exposure than the alternative of regional weather station data.

Despite the above strengths, since effects were measured over the whole study period conclusions cannot be drawn concerning possible changes in these effects over time. It is possible that the effects may differ year to year or between early and late season. Further, to maximize power this study focused on two-day EHEs and was not able to differentiate and assess impacts of rarer more extended EHEs. This limitation leaves a gap in research on the specific impacts of extended EHEs on child health. Similarly, extended hospitalizations may give rise to another limitation due to carryover bias. Specifically, hospitalizations that began during the first week of a month and lasted the entire month would have invalid control days (same day of the week in subsequent weeks) since the child would still be hospitalized. Although this impact cannot be ascertained without access to length of stay data, the rarity of this occurring likely minimizes the potential impact on the overall results. Another limitation is having only examined same day (0-day lag) and 1- and 2-day lagged associations of exposure to 2 consecutive extreme heat days. Though equally short lag periods are common in studies of pediatric morbidity and extreme heat [17, 19, 21–23, 25], some studies have found associations at up to 7-day lag periods [18, 21, 67]. Given that the largest effects were observed at lag 0, declines in effects were observed at lags 1 and 2, and our evaluation of multiple outcomes and exposure measures, we focused on a limited lag period. Further, the time stratified case crossover design of this study uses 3–4 controls days on the same day of week in the same month for each case day. Thus, a 7-day or longer lag period would cause the case and a control day to overlap, potentially biasing results towards the null. Additionally, the ecological design of this study limits generalizability. Individual level factors, like SES and comorbidities, may modify the effect of EHEs on pediatric emergency healthcare use but were not included in this study. Built environments, housing and neighborhood characteristics may affect susceptibility to EHEs within FSAs [68]. Moreover, children who live in remote areas, areas where ED wait times are long, or who have low SES may not be well represented in this study. However, this is unlikely to have systematically biased results since the distribution of urban and rural FSAs of study participants was comparable to the distribution of urban and rural FSAs of children in Ontario, according to the 2011 census [69]. In fact, children with rural FSAs made up a slightly higher proportion (7%) of the distribution within this study than was observed in the 2011 census.

ECCC’s heat alert protocols in Canada identify conditions in which increased all-cause mortality of the general population are likely to occur [2]. In Ontario, regional alert criteria are 2 or more consecutive days with daytime maximum temperatures above 29 °C in Northern Ontario, or 31 °C in South and Southwestern Ontario [4]. These temperatures are most similar to those of the 95th percentile of temperature (see Additional File 1 Table A3). The results of this study may suggest that ECCC’s heat warnings are effective in preventing emergency healthcare use for pediatric injuries as evidenced by the consistently protective effect of EHEs on injuries observed in all analyses, and a contrasting harmful association in adults who are largely unable to change their activities (ex. outdoor labour, commuting to work) [32, 33]. Despite these regional alerts, in analyses using the 95th percentile, EHEs increased rates of pediatric ED visits for heat-related illnesses, heatstroke, dehydration, renal diseases, and otitis; as well as hospital admissions for renal diseases, lower respiratory infections, and bacterial enteritis. It is also important to consider that with increasing intensity and frequency of EHEs due to climate change, today’s 99th percentile temperatures may be the 95th percentile in the near future [2]. Given the suggested efficacy of heat warnings in preventing pediatric injuries, these outcomes may be preventable with more stringent child-specific heat warnings at lower temperatures along with messaging specifying the increased risk of these health impacts.

## Conclusions

Complex relationships were found between EHEs and pediatric emergency healthcare utilization in Ontario. EHEs were positively associated with pediatric hospital admissions due to respiratory illnesses, asthma; infectious and parasitic diseases, lower respiratory infections, and enteritis. EHEs were also positively associated with ED visits due to asthma; heat-related illnesses, heatstroke, dehydration; and lower respiratory infections. Surprisingly, this study found that EHEs had a protective association with injury and transportation-related injury hospital admissions and ED visits.

In wake of a quickly warming climate, it is imperative that heat alert protocols, community emergency planning, urban design, and healthcare facility preparedness be tailored to reflect the impacts of EHEs on child health. Critical gaps in research remain in predicting trends, identifying individual-level vulnerabilities, assessing mental health impacts, and identifying thresholds at which heat impacts child health.

## Electronic supplementary material

Below is the link to the electronic supplementary material.

Supplementary Material 1

Supplementary Material 2

## Data Availability

The health outcome data that support the findings of this study are available from Statistics Canada, but restrictions apply to the availability of these data, which were used under license for the current study, and so are not publicly available. Data are however available from the authors upon reasonable request and with permission of Statistics Canada. The extracted Daymet data used herein are available from the corresponding author on reasonable request.

## Abbreviations

EHE: Extreme heat event
FSA: Forward sortation area
ED: Emergency department
CI: Confidence interval
RR: - Rate ratio
DAD: - Discharge abstract database
NACRS: - National ambulatory care reporting system
WHO: - World health organization
ICD-10: - International statistical classification of disease and related health problems, 10th revision
ECCC: - Environment and climate change canada
SES: - Socioeconomic status
gnm: - Generalized nonlinear model
CIHI: - Canadian institute for health information
