# Supplementary material for: Extreme heat and pediatric health in a warming world: a space-time stratified case-crossover investigation in Ontario, Canada
Source: Environ Health. 2025 Jun 7;24:35. doi: 10.1186/s12940-025-01153-y (PMC12145649; doi:10.1186/s12940-025-01153-y)
Supplement: Supplementary file 1 — Supplementary Material 1 [file 12940_2025_1153_MOESM1_ESM.docx]

# APPENDIX

**Table A1.** Age and sex distributions of pediatric hospital admissions, N= 284,939

|  | **Age in years (n, n/n_T_%)** | | | **Sex (n, n/n_T_%)** | | **Total**  **(n_T_,n_T_/N%)** |
| --- | --- | --- | --- | --- | --- | --- |
|  | 0-4 | 5-12 | 13-18 | Female | Male |  |
| **All-cause** | 133810 | 67175 | 83954 | 130692 | 154233 | 284939 |
|  | 46.96% | 23.58% | 29.46% | 45.87% | 54.13% | 100.00% |
| **Respiratory** | 28095 | 10497 | 4608 | 16630 | 26570 | 43200 |
|  | 65.03% | 24.30% | 10.67% | 38.50% | 61.50% | 15.16% |
| Asthma | 8965 | 5323 | 999 | 5344 | 9943 | 15287 |
|  | 58.64% | 34.82% | 6.53% | 34.96% | 65.04% | 5.37% |
| **Injury** | 10290 | 14484 | 16676 | 15827 | 25622 | 41450 |
|  | 24.83% | 34.94% | 40.23% | 38.18% | 61.81% | 14.55% |
| Drowning | 159 | 108 | 57 | 114 | 210 | 324 |
|  | 49.07% | 33.33% | 17.59% | 35.19% | 64.81% | 0.11% |
| Falls | 4387 | 7451 | 3037 | 5963 | 8912 | 14875 |
|  | 29.49% | 50.09% | 20.42% | 40.09% | 59.91% | 5.22% |
| Transport | 572 | 2831 | 4633 | 2363 | 5672 | 8036 |
|  | 7.12% | 35.23% | 57.65% | 29.41% | 70.58% | 2.82% |
| **Heat** | 1009 | 452 | 259 | 797 | 923 | 1720 |
|  | 58.66% | 26.28% | 15.06% | 46.34% | 53.66% | 0.60% |
| Heatstroke | 8 | 12 | 21 | 16 | 25 | 41 |
|  | 19.51% | 29.27% | 51.22% | 39.02% | 60.98% | 0.01% |
| Dehydration | 1001 | 440 | 238 | 781 | 898 | 1679 |
|  | 59.62% | 26.21% | 14.18% | 46.52% | 53.48% | 0.59% |
| **Renal** | 5959 | 1866 | 1910 | 5902 | 3833 | 9735 |
|  | 61.21% | 19.17% | 19.62% | 60.63% | 39.37% | 3.42% |
| **Inf/Parasitic** | 12415 | 4294 | 2848 | 9072 | 10485 | 19557 |
|  | 63.48% | 21.96% | 14.56% | 46.39% | 53.61% | 6.86% |
| Otitis | 655 | 168 | 65 | 346 | 542 | 888 |
|  | 73.76% | 18.92% | 7.32% | 38.96% | 61.04% | 0.31% |
| Lower resp | 11293 | 2884 | 812 | 6243 | 8746 | 14989 |
|  | 75.34% | 19.24% | 5.42% | 41.65% | 58.35% | 5.26% |
| Enteritis | 6592 | 2304 | 919 | 4523 | 5292 | 9815 |
|  | 67.16% | 23.47% | 9.36% | 46.08% | 53.92% | 3.44% |

**Table A2**. Age and sex distributions of pediatric ED visits, N=5,875,119

|  | **Age in years (n, n/n_T_%)** | | | **Sex (n, n/n_T_%)** | | **Total**  **(n_T_,n_T_/N%)** |
| --- | --- | --- | --- | --- | --- | --- |
|  | 0-4 | 5-12 | 13-18 | Female | Male |  |
| **All-cause** | 2041929 | 1869624 | 1963566 | 2737572 | 3137286 | 5875119 |
|  | 34.76% | 31.82% | 33.42% | 46.60% | 53.40% | 100.00% |
| **Respiratory** | 403339 | 258237 | 180382 | 389705 | 452217 | 841958 |
|  | 47.90% | 30.67% | 21.42% | 46.29% | 53.71% | 14.33% |
| Asthma | 43107 | 45180 | 21151 | 39978 | 69458 | 109438 |
|  | 39.39% | 41.28% | 19.33% | 36.53% | 63.47% | 1.86% |
| **Injury** | 458951 | 710230 | 757570 | 786696 | 1139970 | 1926751 |
|  | 23.82% | 36.86% | 39.32% | 40.83% | 59.17% | 32.80% |
| Drowning | 554 | 494 | 506 | 580 | 974 | 1554 |
|  | 35.65% | 31.79% | 32.56% | 37.32% | 62.68% | 0.03% |
| Falls | 194062 | 243843 | 141251 | 252416 | 326716 | 579156 |
|  | 33.51% | 42.10% | 24.39% | 43.58% | 56.41% | 9.86% |
| Transport | 11921 | 73685 | 94503 | 62869 | 117229 | 180109 |
|  | 6.62% | 40.91% | 52.47% | 34.91% | 65.09% | 3.07% |
| **Heat** | 3401 | 1978 | 2430 | 3776 | 4033 | 7809 |
|  | 43.55% | 25.33% | 31.12% | 48.35% | 51.65% | 0.13% |
| Heatstroke | 311 | 731 | 1039 | 959 | 1122 | 2081 |
|  | 14.94% | 35.13% | 49.93% | 46.08% | 53.92% | 0.04% |
| Dehydration | 3090 | 1247 | 1391 | 2817 | 2911 | 5728 |
|  | 53.95% | 21.77% | 24.28% | 49.18% | 50.82% | 0.10% |
| **Renal** | 33399 | 29830 | 46437 | 92431 | 17232 | 109666 |
|  | 30.46% | 27.20% | 42.34% | 84.28% | 15.71% | 1.87% |
| **Inf/Parasitic** | 192761 | 85780 | 56196 | 162462 | 172259 | 334737 |
|  | 57.59% | 25.63% | 16.79% | 48.53% | 51.46% | 5.70% |
| Otitis | 147926 | 101754 | 33965 | 130044 | 153587 | 283645 |
|  | 52.15% | 35.87% | 11.97% | 45.85% | 54.15% | 4.83% |
| Lower resp | 63522 | 26328 | 11960 | 43346 | 58457 | 101810 |
|  | 62.39% | 25.86% | 11.75% | 42.58% | 57.42% | 1.73% |
| Enteritis | 60263 | 26798 | 15804 | 48072 | 54791 | 102865 |
|  | 58.58% | 26.05% | 15.36% | 46.73% | 53.26% | 1.75% |

**Table A3.** Temperature equivalents of percentiles of maximum daily temperatures by region

| **Location** | **99th percentile** | **97.5th percentile** | **95th percentile** |
| --- | --- | --- | --- |
| **Ontario** | 33.26 | 32.17 | 30.96 |
| **Dfc*** | 32.21 | 30.85 | 29.45 |
| **Dfb*** | 33.26 | 32.17 | 30.96 |
| **Dfa*** | 33.45 | 32.30 | 31.30 |
| **Northern**** | 31.53 | 30.25 | 28.99 |
| **Southern**** | 31.72 | 30.66 | 29.65 |
| **Southwestern**** | 33.30 | 32.23 | 31.03 |
| * Refers to the three Koppen Climate Classifications in which Ontario is situated ** Refers to the three geographic locations in Ontario as decided by the ECCC for use in issuing heat warnings | | | |

| **T****able A4**. Pediatric hospital admissions and EHEs, RR (95%CI) | | | | | | |
| --- | --- | --- | --- | --- | --- | --- |
|  | **Primary Analysis** | **Sensitivity analyses** | | | | |
|  | 99th percentile | Humidity | 1-day lag | 2-day lag | 97.5th percentile | 95th percentile |
| **All Cause** | 1.05 (0.98, 1.12) | 1.05 (0.98, 1.12) | 1.02 (0.95, 1.09) | **1.08 (1.00, 1.15)** | **1.04 (1.00, 1.07)** | 1.01 (0.98, 1.03) |
| **Respiratory** | **1.26 (1.14, 1.40)** | **1.26 (1.14, 1.39)** | 1.00 (0.90, 1.12) | 0.92 (0.82, 1.03) | **1.18 (1.12, 1.24)** | 1.01 (0.98, 1.04) |
| Asthma | **1.29 (1.16, 1.44)** | **1.29 (1.16, 1.45)** | 1.01 (0.90, 1.14 | *0.73 (0.64, 0.83)* | **1.08 (1.02, 1.15)** | 0.96 (0.93, 1.00) |
| **Injury** | *0.87 (0.79, 0.96)* | *0.85 (0.77, 0.93)* | *0.89 (0.81, 0.98)* | *0.79 (0.71, 0.86)* | *0.93 (0.89, 0.98)* | 0.99 (0.96, 1.02) |
| Drowning* | 1.07 (0.98, 1.17) | **1.10 (1.00, 1.20)** | *0.52 (0.48, 0.57)* | *0.00 (0.00, 0.70)* | *0.88 (0.85, 0.92)* | **1.98 (1.94, 2.03)** |
| Falls | 0.93 (0.84, 1.04) | 0.89 (0.80, 1.00) | 0.97 (0.88, 1.07) | *0.69 (0.62, 0.77)* | 0.97 (0.92, 1.02) | 1.03 (0.99, 1.06) |
| Transport | *0.87 (0.79, 0.96)* | *0.81 (0.73, 0.89)* | *0.57 (0.51, 0.63)* | *0.42 (0.38, 0.48)* | *0.88 (0.83, 0.92)* | *0.95 (0.92, 0.98)* |
| **Heat*** | **1.44 (1.33, 1.56)** | **1.44 (1.34, 1.56)** | **1.87 (1.73, 2.03)** | **1.65 (1.47, 1.84)** | **1.32 (1.27, 1.38)** | **1.41 (1.37, 1.45)** |
| Heatstroke* | **2.39E5 (1.11E3, 5.13E7)** | **3.03E5 (1.86E3, 5.00E7)** | **2.38E5 (1.11E2, 5.12E8)** | **2.38E5 (1.11E2, 5.13E8)** | **6.50E5 (5.01E3, 8.43E7)** | **21.00 (20.39, 21.63)** |
| Dehydration* | 1.08 (0.99, 1.18) | **1.09 (1.00, 1.19)** | **1.65 (1.51, 1.79)** | **1.24 (1.10, 1.40)** | 1.00 (0.95, 1.05) | **1.24 (1.20, 1.28)** |
| **Renal** | 0.94 (0.86, 1.04) | 0.95 (0.87, 1.05) | 0.90 (0.81, 1.00) | **1.56 (1.42, 1.72)** | **1.06 (1.01, 1.11)** | **1.13 (1.09, 1.16)** |
| **Inf/Parasitic** | **1.36 (1.24, 1.50)** | **1.37 (1.24, 1.51)** | **1.18 (1.07, 1.29)** | **1.39 (1.26, 1.53)** | **1.10 (1.05, 1.16)** | 1.02 (0.98, 1.05) |
| Otitis* | *0.00 (0.00, 0.20)* | *0.00 (0.00, 0.18)* | *0.86 (0.78, 0.95)* | **1.33 (1.20, 1.48)** | *0.49 (0.46, 0.52)* | *0.52 (0.50, 0.55)* |
| Lower resp | **1.50 (1.36, 1.67)** | **1.51 (1.36, 1.67)** | **1.32 (1.18, 1.48)** | 0.97 (0.86, 1.09) | **1.34 (1.27, 1.41)** | **1.07 (1.04, 1.11)** |
| Enteritis | **1.19 (1.07, 1.32)** | **1.19 (1.07, 1.32)** | **1.32 (1.19, 1.46)** | 1.10 (0.98, 1.24) | **1.18 (1.12, 1.24)** | **1.10 (1.06, 1.13)** |
| * Occurrence was very rare with less than 20 admissions during EHEs throughout the study period. Consequently, results for these outcomes were imprecise and so were not presented in main study  Numeric values in bold font represent a statistically significant positive association  Numeric values in italicised font represent a statistically significant negative association  To view plot visualizations of the data in this table, visit: (1) <https://hallah-kassem.shinyapps.io/pediatric_health_impacts_of_heat/> | | | | | | |

| **Table A5**. Pediatric emergency department visits and EHEs, RR (95%CI) | | | | | | |
| --- | --- | --- | --- | --- | --- | --- |
|  | **Primary Analysis** | **Sensitivity analyses** | | | | |
|  | 99th percentile | Humidity | 1-day lag | 2-day lag | 97.5th percentile | 95th percentile |
| **All Cause** | 0.98 (0.96, 1.00) | *0.97 (0.95, 0.99)* | 1.00 (0.98, 1.02) | 1.01 (0.99, 1.03) | *0.99 (0.98, 0.99)* | *0.99 (0.98, 0.99)* |
| **Respiratory** | 0.96 (0.92, 1.01) | 0.96 (0.91, 1.01) | *0.95 (0.90, 0.99)* | 0.97 (0.93, 1.02) | 0.99 (0.96, 1.01) | *0.98 (0.96, 0.99)* |
| Asthma | **1.18 (1.07, 1.29)** | **1.18 (1.07, 1.29)** | 0.99 (0.90, 1.09) | *0.90 (0.81, 0.99)* | **1.07 (1.02, 1.12)** | 1.00 (0.97, 1.03) |
| **Injury** | *0.94 (0.91, 0.97)* | *0.92 (0.88, 0.95)* | *0.93 (0.90, 0.96)* | *0.92 (0.89, 0.95)* | *0.94 (0.92, 0.95)* | *0.95 (0.94, 0.96)* |
| Drowning* | **1.15 (1.05, 1.26)** | 1.07 (0.98, 1.18) | *0.58 (0.53, 0.63)* | 1.02 (0.95, 1.09) | *0.86 (0.82, 0.90)* | **1.31 (1.27, 1.34)** |
| Falls | *0.91 (0.85, 0.96)* | *0.88 (0.83, 0.93)* | *0.90 (0.85, 0.95)* | *0.90 (0.85, 0.96)* | *0.91 (0.88, 0.94)* | *0.92 (0.90, 0.93)* |
| Transport | *0.87 (0.80, 0.94)* | *0.82 (0.76, 0.88)* | *0.77 (0.72, 0.84)* | *0.86 (0.80, 0.92)* | *0.85 (0.82, 0.88)* | *0.90 (0.88, 0.92)* |
| **Heat** | **3.11 (2.93, 3.30)** | **2.95 (2.79, 3.13)** | **2.44 (2.28, 2.61)** | **1.78 (1.66, 1.91)** | **3.17 (3.07, 3.28)** | **2.62 (2.56, 2.68)** |
| Heatstroke | **6.90 (6.60, 7.22)** | **5.96 (5.69, 6.24)** | **4.38 (4.16, 4.62)** | **1.53 (1.44, 1.63)** | **7.07 (6.88, 7.26)** | **5.18 (5.09, 5.27)** |
| Dehydration | **1.35 (1.25, 1.46)** | **1.33 (1.23, 1.44)** | **1.56 (1.44, 1.69)** | **1.94 (1.80, 2.10)** | **1.57 (1.51, 1.64)** | **1.44 (1.40, 1.48)** |
| **Renal** | 1.04 (0.96, 1.13) | 1.05 (0.97, 1.14) | **1.08 (1.00, 1.18)** | 1.02 (0.94, 1.10) | **1.04 (1.00, 1.08)** | **1.03 (1.01, 1.06)** |
| **Inf/Parasitic** | 1.00 (0.93, 1.07) | 0.99 (0.93, 1.06) | 1.00 (0.94, 1.07) | 0.98 (0.92, 1.04) | 0.98 (0.95, 1.01) | 1.00 (0.97, 1.02) |
| Otitis | 1.02 (0.96, 1.07) | 1.02 (0.97, 1.08) | **1.19 (1.13, 1.26)** | **1.44(1.37, 1.52)** | 1.03 (0.99, 1.08) | **1.05 (1.03, 1.07)** |
| Lower resp | **1.10 (1.00, 1.21)** | **1.09 (1.00, 1.20)** | 1.05 (0.96, 1.15) | *0.91 (0.83, 0.99)* | 1.03 (0.99, 1.08) | 1.00 (0.97, 1.03) |
| Enteritis | 0.93 (0.84, 1.02) | 0.93 (0.84, 1.02) | **1.10 (1.01, 1.20)** | 0.96 (0.88, 1.05) | 0.98 (0.93, 1.02) | 1.02 (0.99, 1.05) |
| * Occurrence was very rare with less than 20 ED visits during EHEs throughout the study period. Consequently, results for these outcomes were imprecise and so were not presented in main study  Numeric values in bold font represent a statistically significant positive association  Numeric values in italicised font represent a statistically significant negative association  To view plot visualizations of the data in this table, visit: (1) <https://hallah-kassem.shinyapps.io/pediatric_health_impacts_of_heat/> | | | | | | |

##### **Table A6**. Hospital admissions primary analysis by age category, RR (95%CI)

|  | **Age in years** | | |
| --- | --- | --- | --- |
|  | 0-4 | 5-12 | 13-18 |
| **All Cause** | 1.05 (0.97, 1.14) | 0.97 (0.89, 1.07) | **1.20 (1.01, 1.20)** |
| **Respiratory** | **1.19 (1.07, 1.32)** | 0.93 (0.82, 1.07) | **2.19 (2.02, 2.38)** |
| Asthma | **1.38 (1.24,1.53)** | *0.81 (0.69, 0.94)* | **2.24 (2.05, 2.45)** |
| **Injury** | **1.13 (1.04, 1.24)** | *0.75 (0.68, 0.84)* | *0.81 (0.73, 0.90)* |
| Drowning | 1.00 (0.00, 1.40E75) | *0.00 (0.00, 0.34)* | **1.65 (1.58, 1.71)** |
| Falls | **1.39 (1.27, 1.52)** | *0.62 (0.54, 0.71)* | 0.92 (0.83, 1.02) |
| Transport | *0.84 (0.77, 0.91)* | *0.73 (0.66, 0.81)* | 0.97 (0.88, 1.07) |
| **Heat** | **1.27 (1.18, 1.38)** | **2.00 (1.84, 2.18)** | **1.65 (1.51, 1.79)** |
| Heatstroke | **6.40E5 (4.16E3, 9.87E7)** | **6.42e5 (8.01E2, 5.14E8)** | 1.00 (0.00, 1.75E74) |
| Dehydration | 1.02 (0.94, 1.10) | 1.00 (0.90, 1.11) | **1.65 (1.52, 1.79)** |
| **Renal** | *0.81 (0.73, 0.89)* | *0.83 (0.74, 0.94)* | **1.37 (1.27, 1.48)** |
| **Inf/Parasitic** | **1.11 (1.01, 1.24)** | **3.66 (3.33, 4.03)** | *0.69 (0.62, 0.77)* |
| Otitis | *0.00 (0.00, 0.46)* | *0.00 (0.00, 0.59)* | *0.00 (0.00, 0.13)* |
| Low resp | **1.58 (1.43, 1.74)** | *0.80 (0.70, 0.91)* | **4.77 (4.26, 5.35)** |
| Enteritis | *0.53 (0.46, 0.62)* | **5.04 (4.61, 5.51)** | *0.45 (0.41, 0.50)* |
| Numeric values in bold font represent a statistically significant positive association  Numeric values in italicised font represent a statistically significant negative association | | | |

##### **Table A7**. ED visits primary analysis by age category, RR (95%CI)

|  | **Age in years** | | |
| --- | --- | --- | --- |
|  | 0-4 | 5-12 | 13-18 |
| **All Cause** | 0.97 (0.94, 1.01) | *0.96 (0.93, 0.99)* | 1.02 (0.98, 1.05) |
| **Respiratory** | 0.99 (0.92, 1.05) | *0.89 (0.83, 0.95)* | 1.04 (0.97, 1.12) |
| Asthma | **1.30 (1.18, 1.44)** | 1.00 (0.90, 1.11) | **1.33 (1.20, 1.47)** |
| **Injury** | *0.93 (0.88, 0.99)* | *0.88 (0.83, 0.92)* | 1.00 (0.97, 1.05) |
| Drowning | 1.07 (0.95, 1.21) | *0.00 (0.00, 0.24)* | **2.24 (2.10, 2.39)** |
| Falls | 0.93 (0.86, 1.00) | *0.85 (0.78, 0.92)* | 0.97 (0.89, 1.06) |
| Transport | *0.76 (0.68, 0.85)* | *0.78 (0.72, 0.86)* | 0.94 (0.87, 1.02) |
| **Heat** | **1.74 (1.62, 1.86)** | **4.63 (4.37, 4.91)** | **3.92 (3.72, 4.13)** |
| Heatstroke | **4.29 (4.14, 4.44)** | **10.48 (9.92, 11.08)** | **7.05 (6.72, 7.39)** |
| Dehydration | 0.97 (0.89, 1.07) | **1.83 (1.70, 1.97)** | **1.65 (1.54, 1.76)** |
| **Renal** | **1.25 (1.14, 1.37)** | *0.81 (0.73, 0.90)* | 1.04 (0.96, 1.14) |
| **Inf/Parasitic** | 0.99 (0.92, 1.07) | 0.94 (0.85, 1.03) | **1.12 (1.02, 1.23)** |
| Otitis | 0.98 (0.91, 1.06) | 1.06 (0.99, 1.12) | 1.00 (0.93, 1.07) |
| Low resp | **1.14 (1.03, 1.26)** | 0.90 (0.81, 1.01) | **1.43 (1.28, 1.61)** |
| Enteritis | *0.85 (0.77, 0.94)* | 0.92 (0.82, 1.03) | **1.27 (1.15, 1.42)** |
| Numeric values in bold font represent a statistically significant positive association  Numeric values in italicised font represent a statistically significant negative association | | | |

##### **Table A8**. Hospital admissions primary analysis by sex, RR (95%CI)

|  | **Sex (n, n/n_T_%)** | |
| --- | --- | --- |
|  | Female | Male |
| **All Cause** | 1.00 (0.93, 1.09) | **1.08 (1.00, 1.17)** |
| **Respiratory** | **1.27 (1.14, 1.41)** | **1.26 (1.14, 1.40)** |
| Asthma | **1.21 (1.08, 1.36)** | **1.33 (1.20, 1.50)** |
| **Injury** | **1.17 (1.06, 1.29)** | *0.72 (0.65, 0.80)* |
| Drowning | **1.75 (1.56, 1.74)** | 0.00 (0.00, 9.86) |
| Falls | 1.04 (0.94, 1.16) | *0.86 (0.77, 0.96)* |
| Transport | **1.50 (1.38, 1.63)** | *0.65 (0.58, 0.72)* |
| **Heat** | **1.22 (1.14, 1.32)** | **1.76 (1.62, 1.91)** |
| Heatstroke | **6.47E5 (3.07E3, 1.36E8)** | 1.00 (0.00, 1.57E73) |
| Dehydration | *0.61 (0.56, 0.67)* | **1.76 (1.62, 1.91)** |
| **Renal** | 1.08 (0.99, 1.18) | *0.74 (0.66, 0.82)* |
| **Inf/Parasitic** | **1.34 (1.20, 1.50)** | **1.37 (1.26, 1.50)** |
| Otitis | *0.00 (0.00, 0.18)* | *0.00 (0.00, 0.22)* |
| Low resp | **1.65 (1.48, 1.83)** | **1.42 (1.28, 1.57)** |
| Enteritis | **1.14 (1.01, 1.29)** | **1.22 (1.10, 1.34)** |
| Numeric values in bold font represent a statistically significant positive association  Numeric values in italicised font represent a statistically significant negative association | | |

##### **Table A9.** ED visits primary analysis by sex, RR (95%CI)

|  | **Sex (n, n/n_T_%)** | |
| --- | --- | --- |
|  | Female | Male |
| **All Cause** | 0.99 (0.96, 1.02) | 0.98 (0.95, 1.00) |
| **Respiratory** | 0.98 (0.92, 1.04) | 0.95 (0.89, 1.02) |
| Asthma | **1.30 (1.17, 1.43)** | **1.11 (1.01, 1.23)** |
| **Injury** | *0.91 (0.86, 0.95)* | 0.96 (0.92, 1.00) |
| Drowning | *0.00 (0.00, 0.57)* | **1.81 (1.67, 1.95)** |
| Falls | *0.86 (0.79, 0.93)* | 0.94 (0.88, 1.01) |
| Transport | 0.95 (0.87, 1.04) | *0.82 (0.76, 0.90)* |
| **Heat** | **2.47 (2.33, 2.61)** | **4.00 (3.76, 4.25)** |
| Heatstroke | **6.15 (5.90, 6.40)** | **8.06 (7.65, 8.49)** |
| Dehydration | *0.67 (0.60, 0.73)* | **2.26 (2.10, 2.43)** |
| **Renal** | 1.04 (0.96, 1.13) | 1.06 (0.96, 1.16) |
| **Inf/Parasitic** | 1.01 (0.93, 1.09) | 0.99 (0.91, 1.07) |
| Otitis | **1.11 (1.05, 1.19)** | 0.93 (0.87, 1.00) |
| Low resp | 1.07 (0.97, 1.19) | **1.12 (1.01, 1.24)** |
| Enteritis | 0.98 (0.88, 1.09) | *0.88 (0.79, 0.98)* |
| Numeric values in bold font represent a statistically significant positive association  Numeric values in italicised font represent a statistically significant negative association | | |
