# Supplementary material for: Extreme heat and pediatric health in a warming world: a space-time stratified case-crossover investigation in Ontario, Canada
Source: Environ Health. 2025 Jun 7;24:35. doi: 10.1186/s12940-025-01153-y (PMC12145649; doi:10.1186/s12940-025-01153-y)
Supplement: Supplementary file 2 — Supplementary Material 2 [file 12940_2025_1153_MOESM2_ESM.pptx]

## Slide 1
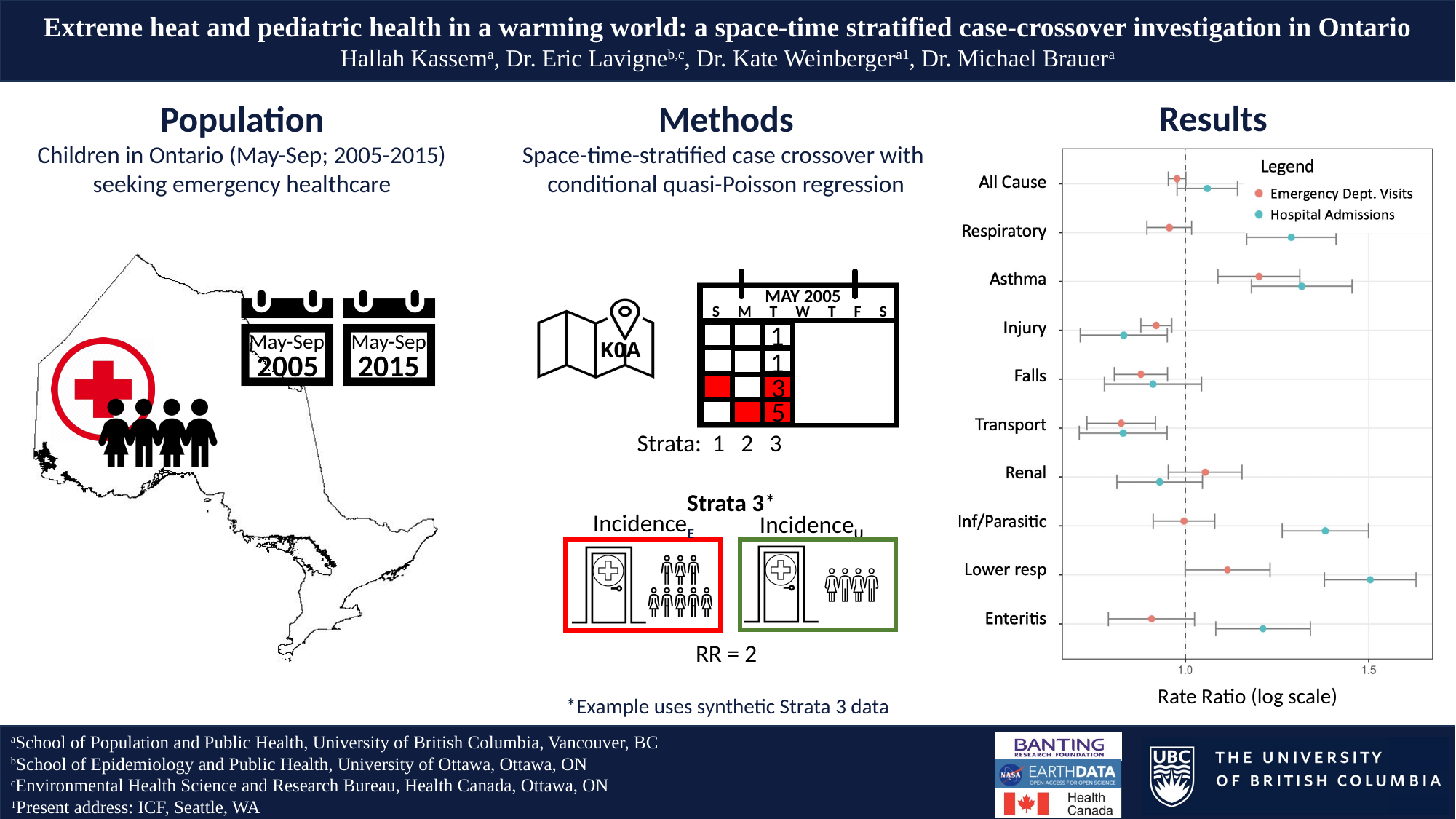

Extreme heat and pediatric health in a warming world: a space-time stratified case-crossover investigation in Ontario
Hallah Kassema, Dr. Eric Lavigneb,c, Dr. Kate Weinbergera1, Dr. Michael Brauera
Results
Population
Children in Ontario (May-Sep; 2005-2015) seeking emergency healthcare
Methods
Space-time-stratified case crossover with
conditional quasi-Poisson regression
Rate Ratio (log scale)
MAY 2005
S M T W T F S
1
May-Sep
May-Sep
K0A
1
2005
2015
3
5
Strata: 1 2 3
Strata 3*
IncidenceE
IncidenceU
RR = 2
*Example uses synthetic Strata 3 data
aSchool of Population and Public Health, University of British Columbia, Vancouver, BC
bSchool of Epidemiology and Public Health, University of Ottawa, Ottawa, ON
cEnvironmental Health Science and Research Bureau, Health Canada, Ottawa, ON
1Present address: ICF, Seattle, WA
